# Hepatogenous photosensitisation in cows grazing turnips (*Brassica rapa*) in South Africa

**DOI:** 10.4102/jsava.v92i0.2106

**Published:** 2021-05-06

**Authors:** Anthony J. Davis, Mark G. Collet, Johan C.A. Steyl, Jan G. Myburgh

**Affiliations:** 1 Humansdorp Veterinary Clinic, Humansdorp, South Africa; 2 School of Veterinary Science, Massey University, Palmerston North, New Zealand; 3 Department of Paraclinical Sciences, Faculty of Veterinary Science, University of Pretoria, Onderstepoort, South Africa

**Keywords:** *Brassica rapa*, forage turnip, dairy cattle, teat lesions, Barkant, *Brassica*-associated liver disease, hepatogenous photosensitivity, South Africa

## Abstract

Holstein cows on a farm in the Humansdorp district, Eastern Cape province, South Africa, developed reddened, painful teat skin 3 days after grazing a mixed forage crop dominated by bulb turnip (*Brassica rapa*, Barkant cultivar). The crop was grazed 45 days after planting and 10% of the herd developed symptoms. More characteristic non-pigmented skin lesions started manifesting 1–2 days after the appearance of the teat lesions. Affected cows had elevated serum activities of gamma-glutamyl transferase, glutamate dehydrogenase and aspartate aminotransferase. These blood chemistry findings confirmed a secondary (hepatogenous) photosensitivity. As a result of the severity of the teat and skin lesions, seven cows were slaughtered and tissue samples from five of them were collected for histopathological examination. Liver lesions in cows that were culled 3 or more weeks after the onset of the outbreak showed oedematous concentric fibrosis around medium-sized bile ducts and inflammatory infiltrates in portal tracts. Characteristic lesions associated with other known hepatobiliary toxicities were not found. No new cases were reported 5 days after the cattle were removed from the turnips. The sudden introduction of the cows, without any period of transitioning or adaptation to grazing turnips, as well as the short latent period, clinical signs of photosensitisation, blood chemistry and histopathology, confirmed a diagnosis of *Brassica*-associated liver disease, a condition seen in New Zealand but not previously described in South Africa. *Brassica* forage crops are potentially toxic under certain conditions and farmers must be aware of these risks.

## Introduction

There are several causes of hepatogenous or secondary photosensitisation in ruminants in South Africa (Kellerman & Coetzer 1985). In addition to various hepatotoxic plants, there are water-borne cyanobacteria, such as *Microcystis aeruginosa*, which cause acute clinical signs and high mortality (Kellerman & Coetzer 1985). A mycotoxin, sporidesmin, found in spores produced by the saprophytic fungus *Pithomyces chartarum*, has also been described (Kellerman et al. 2005). These poisonous agents are classified into hepatocellular toxins (e.g. those produced by the cyanobacteria) and biliary system toxins (e.g. sporidesmin, the cause of facial eczema [FE]), dependent on their predominant mechanism of action (Kellerman et al. 2005).

In cattle, hepatogenous photosensitivity associated with the feeding of crops comprising certain cultivars and/or hybrids of forage *Brassica*, namely turnip (*Brassica rapa* ssp. *rapa*), rape (*B. napus* ssp. *biennis*) and swedes (rutabaga, *B. napus* ssp. *napobrassica*) has been reported from Australia (Morton & Campbell 1997) and New Zealand (Collett & Matthews 2014). This bovine clinical entity is now referred to as *Brassica*-associated liver disease (BALD) (Matthews, Parton & Collett 2020). The incidence, clinical signs, blood chemical and histological changes were first described by Collett and Matthews (2014). The Brassicaceae family of plants is also associated with various well-known clinical syndromes, including haemolytic anaemia, polioencephalomalacia, abortion, nitrate toxicity, ruminal acidosis, constipation or diarrhoea, goitre, fog fever and bloat as a result of choke, as well as the problem of *Brassica* milk taint (Cote 1944; Westwood & Nichol 2009; Wikse, Leathers & Parish 1987).

The hepatotoxin causing BALD has not yet been identified, but nitrile derivatives of certain glucosinolates, the sulphur-containing secondary compounds found in all *Brassica* spp., are suspected to play a role (Collett, Stegelmeier & Tapper 2014; Matthews et al. 2020). The unknown toxin affects liver parenchymal tissue and small bile ducts, resulting in secondary photosensitivity (Collett 2014). In some cows, skin lesions are mild and confined to the teats and limited areas of non-pigmented skin. In others, more severe and extensive non-pigmented skin lesions, characterised by epidermal and superficial dermal necrosis leading to sloughing, as well as oedema of the extremities may lead to dehydration and death or necessitate culling on welfare grounds (Collett & Matthews 2014).

In New Zealand, camps of these high-yielding *Brassica* forage crops are planted to supplement nutritional requirements when pasture growth is slow or of poor quality (Westwood & Nichol 2009). In recent years, such crops have been introduced to the Eastern Cape Province to fill a forage gap, for example, in the transition between pastures or when feed supplies are limited.

## Outbreak history and clinical findings

The outbreak occurred on a farm located about 30 km south-east of Humansdorp and about 4 km from the coast of the Eastern Cape Province. In December 2018, the area was experiencing hot and dry conditions and the availability of good quality feed was becoming scarce. Perennial ryegrass (*Lolium perenne*) and kikuyu (*Pennisetum clandestinum*) pastures had been heavily grazed and supplies of perennial ryegrass hay were limited. After being fed approximately 14 kg ryegrass silage per cow for 7 days, the herd of approximately 400 lactating, mostly in-calf, registered Holstein cows was abruptly introduced (Day 0) to a camp that had been planted 45 days previously with a seed mix comprising of four forage species, namely bulb turnip (*B. rapa*, Barkant cultivar), rape (*B. napus*, Essex Giant English cultivar), forage sorghum (a hybrid of *S. saccharatum* and *S. sudanense* called Big Kahuna) and sunhemp (*Crotalaria intermedia*). At night, the cows grazed a pasture mix of kikuyu and ryegrass.

**FIGURE 1:**
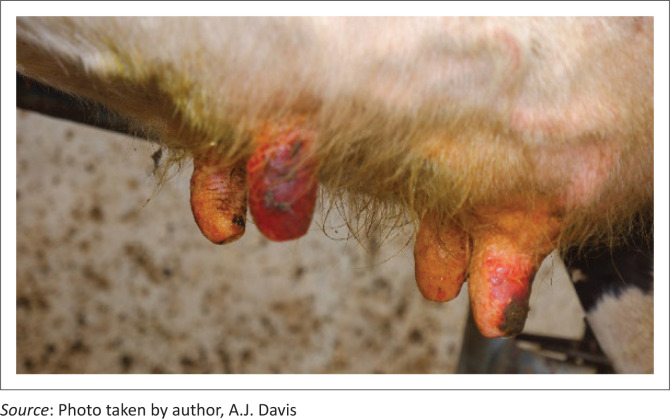
Teats of a cow with acute photosensitisation showing reddening, erosions and crusting.

On the evening of Day 3, milkers noticed that approximately 20 cows had sensitive and painful teats. The next morning, these cows refused to be milked. There were erosions on affected teat skin accompanied by a serous discharge. On Day 5 (Monday, 1st January 2019), six affected cows (Cases 1–6) were presented for clinical examination. One had brisket oedema and another showed swollen distal forelimbs. The white-haired areas of the coat appeared normal. Body temperatures were increased (39.8 °C – 40.5 °C) and heart rates were elevated (88–100/min). Rumen contractions were normal. Mucous membranes were dark pink. Habitus was good, except for painful teat lesions (Figure 1) and abrasions on the skin of the udder. At this stage, differential diagnoses included photosensitisation (although non-pigmented skin excluding the udder appeared normal), a contact irritant from a possibly contaminated teat dip, a viral or bacterial infection of the teats or a vasoconstrictive disease, for example, ergotism.

**FIGURE 2:**
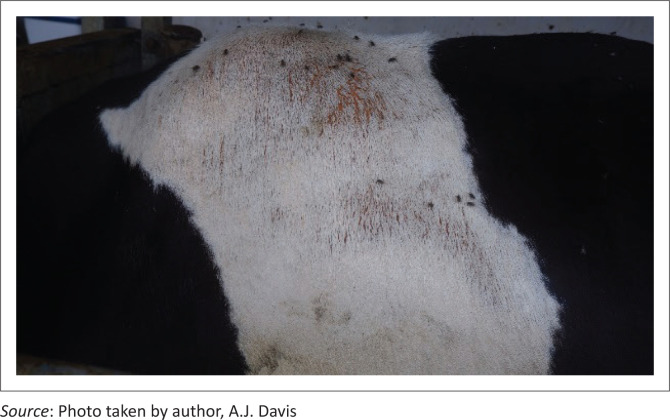
Non-pigmented (white) skin of a Holstein cow with photosensitisation showing reddening and oozing of serum.

**FIGURE 3:**
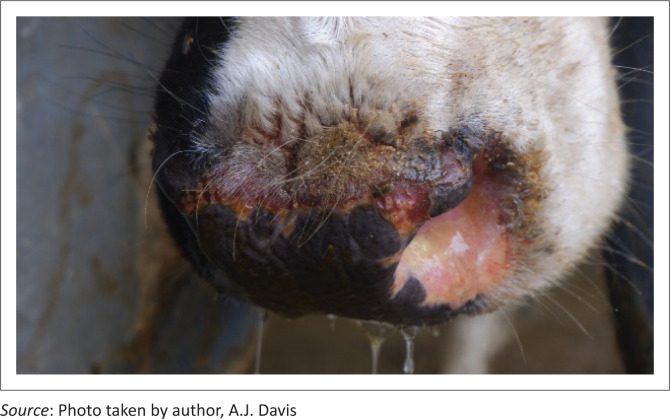
Dorsal muzzle of a cow with photosensitisation showing erosions, ulcers, fissures and crusts in the adjacent non-pigmented skin.

The next day (Day 6), the farmer reported that an additional 21–30 cows had teat sensitivity and other signs of early photosensitivity (hyperaemia and lichenification on non-pigmented areas of skin; Figure 2). In addition, clinical signs noted in some cows included hypersalivation and erosions, ulcers, fissures, crusts and serous exudation of non-pigmented skin adjacent to the muzzle (Figure 3), vulva and eyelids.

### Clinical pathology

On Day 5, blood samples were collected from the caudal tail vein into Beckton, Dickinson (BD) Vacutainer® serum tubes for blood chemistry (Cases 1–6) and BD Vacutainer® EDTA tubes for routine haematology (Cases 1–3). All six cows had lesions on the teats only. These were submitted to Pathcare Laboratories, Jeffrey’s Bay. Clear evidence of hepatic injury was demonstrated by the elevated activities of gamma-glutamyl transferase (GGT, mean 563 international unit per litre) IU/L, range 430 IU/L – 842 IU/L, laboratory reference range 0 IU/L – 20 IU/L) and aspartate aminotransferase (AST, mean 286 IU/L, range 183 IU/L – 537 IU/L, reference range 18 IU/L – 153 IU/L). On complete blood count all parameters were within normal limits except for two of the three animals tested that showed a mild neutrophilia. No blood-borne parasites could be detected on routine blood smear (Diff Quik®) examination.

On Day 8, blood samples for serum chemistry were collected from a further six cows (Cases 7–12) and submitted to the Clinical Pathology Laboratory, Faculty of Veterinary Science, University of Pretoria, Onderstepoort. All six had severe lesions on the teats, five had mild lesions on the muzzle and three had moderate to severe lesions on non-pigmented skin. As before, hepatic injury was indicated by elevated activities of GGT (mean 267 IU/L, range 225 IU/L – 394 IU/L, reference range 0 IU/L – 20 IU/L), AST (mean 155 IU/L, range 97 IU/L – 196 IU/L, reference range 33 IU/L – 95 IU/L) and glutamate dehydrogenase (GDH, mean 199 IU/L, range 43 IU/L – 604 IU/L, reference range 5 IU/L – 14 IU/L).

### Treatment

Topical acriflavine-glycerine treatment of reddened, sensitive teats relieved the inflammation and cows tolerated the teat clusters better at the next milking 12 h later. Additional supportive therapy included an intramuscular injection of 30 mL of long-acting penicillin (Duplocillin®, MSD), repeated after 2 days, a single subcutaneous injection of 12 mL of an anti-inflammatory drug (Metacam®, Boehringer Ingelheim) and an intramuscular injection of 20 mL once daily for 3 days of thioctic acid (a liver stimulant, Tioctan Vet®, Bayer, Germany; MSD Animal Health, Kempton Park, South Africa; Kyron Laboratories (Pty) Ltd, Johannesburg, South Africa) and 15 mL of vitamin C (KyroviteC®, Kyron Laboratories) once daily for 3–5 days. However, cows with severely affected teats (reddened, ulcerated and painful) could not be milked as the teat clusters aggravated the irritation and hand-milking was equally painful. These cows were in full production and could not be dried off at this point and this necessitated slaughter of at least two cows.

### On farm investigation

The mixed forage crop that the cows had been grazing was examined. The dominant component was the Barkant turnip and it had been heavily grazed. The English Giant rape had very hard leaves and the cows ate less of this crop. The sorghum was a minor component and sunhemp was very sparse. Very few weeds were present, but insect damage was apparent in the *Brassicas*. According to the farm manager, the crop had been fertilised only once, at the time of planting. During each day that the cows were on the crop, they had unrestricted access until the afternoon milking after which they were kept on a ryegrass/kikuyu pasture overnight. No hay was fed during this period.

The crop and neighbouring pastures were inspected for plants known to cause either secondary or primary photosensitivity in the Eastern Cape Province, namely *Lantana camara, Lasiospermum bipinnatum, Athanasia trifurcata* (Kellerman & Coetzer 1985) and *Ammi majus* (Van Niekerk 1994), but none were found. There was also no evidence of algal blooms in any of the water sources.

The cows were removed from the forage crop on Day 8. By Day 13, no new cases were seen in the herd. A total of seven cows had to be slaughtered for welfare reasons as a result of the severity of the lesions.

### Mycology

Samples of the *Brassica* leaf and bulb material and the ryegrass hay were collected and examined for the presence of *P. chartarum* spores using the method of Di Menna and Bailey (1973). Leaf washing and spore counts were performed on the same day of collection.

*Pithomyces chartarum* spore counts of more than 200 000 spores/g are considered potentially toxic (Di Menna & Bailey 1973). Although spores resembling *P. chartarum* were seen, counts were exceedingly low (< 5000 spores/g).

Fungal cultures of *Brassica* plant material revealed *Fusarium* spp. on the bulbs and leaves, *Cladosporium* spp. from the leaf debris. No *P. chartarum* was cultured from this plant material (Capp, E, University of Pretoria).

### Necropsies and histopathology

Four cows that had severe teat lesions which exhibited extreme distress during milking were culled on Day 6. Macroscopically, no significant lesions were observed apart from the previously described dermal changes on clinical examination. Liver, gallbladder and kidney from two of these cows (Cases 2 and 6, GGT activities 463 IU/L and 525 IU/L, respectively) were collected in 10% neutral buffered formalin for histopathological examination.

Microscopically, portal tracts were oedematous with infiltrations of moderate numbers of mononuclear cells and fewer neutrophils and multifocal microabscesses occurred periportally. Early bile duct hyperplasia and portal tract fibrosis were also noted.

**FIGURE 4:**
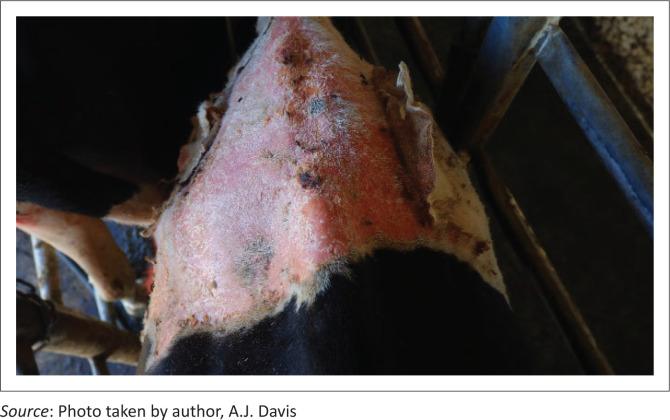
Cow (Case 3) with extensive lesions of photosensitisation of the non-pigmented (white) skin of the withers and dorsal thorax 5 weeks after grazing turnips.

A fifth cow (Case 13) was culled and sampled for histology only on Day 21. As a result of sloughing of extensive areas of non-pigmented skin, a further two cows (Cases 3 and 14) were culled and sampled 5 weeks after signs were first seen (Figure 4). Case 3 had a GGT activity of 526 IU/L on Day 5 and at slaughter 156 IU/L, whilst Case 14, sampled at slaughter only, had a GGT of 332 IU/L. The livers of Cases 13, 3 and 14 showed subtle mottling, were not noticeably misshapen and the gallbladder wall showed a few small haemorrhages. Samples of liver (from multiple lobes), gallbladder, kidney, skin, rumen, abomasum, small intestine, lung, heart, spleen, lymph node and tongue were fixed in formalin for histopathological examination.

**FIGURE 5:**
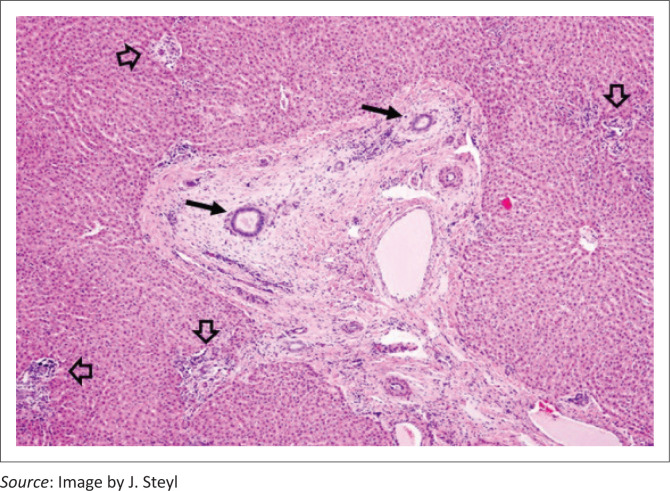
Photomicrograph of the liver of Case 14, a cow that was still photosensitive 5 weeks after grazing turnips. There is loose, oedematous collagenous mesenchymal tissue surrounding medium-sized bile ducts (black arrows) whilst surrounding small portal triads show mild lymphocytic infiltration (open arrows) (haematoxylin and eosin [H&E] × 4).

**FIGURE 6:**
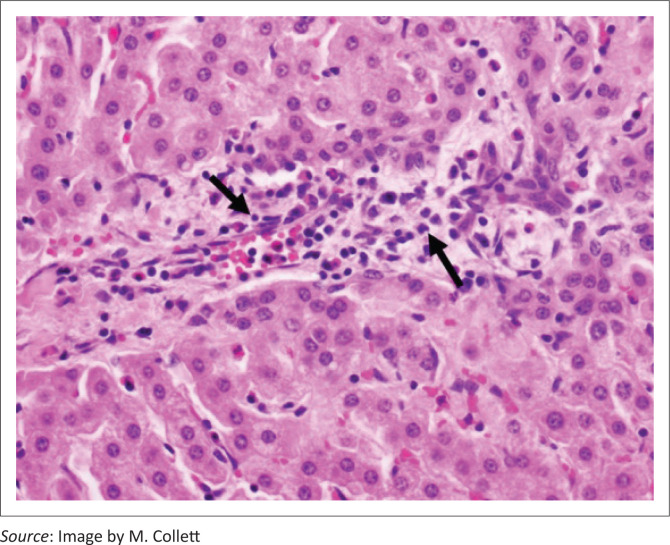
High power photomicrograph of the liver of Case 14: A cow that was still photosensitive 5 weeks after grazing turnips. A small portal tract is infiltrated with lymphocytes (arrows) and isolated neutrophils and lacks a recognisable bile duct (haematoxylin and eosin [H&E] × 40).

Liver lesions in these three cases were similar. The most conspicuous lesion, easily visible at low power microscopic examination, and mainly restricted to the medium to large portal tracts, was mild to moderate loose (oedematous), concentric (onion skin), periductular biliary fibroplasia (Figure 5) associated with biliary epithelial hypertrophy, mild occasional bile duct proliferation and random single cell biliary epithelial necrosis. Smaller portal tracts were infiltrated with low to moderate numbers of mononuclear cells, fewer degenerate neutrophils and scant eosinophils that frequently obscured the components of the portal triad (Figure 6). Mild bridging fibrosis was confined to only small areas in a lobe.

The skin of Case 13 showed severe diffuse epidermal corneal mixed inflammatory exudation (serum, neutrophils and macrophages) and orthokeratosis, mild acanthosis and apocrine tubular ectasia, as well as subepidermal fibrosis with mild eosinophilia and lymphocytosis. The skin of Cases 3 and 14 showed mild orthokeratotic acanthosis and moderate diffuse subepidermal fibroplasia. Other examined tissues were unremarkable.

## Discussion

Most of the plants known to cause photosensitivity do not occur in this area of the Eastern Cape, with the exception of *Lantana camara*, a common invasive exotic causing secondary photosensitivity. *Ammi majus* has been associated with primary photosensitivity and contact dermatitis of the teats and udder skin in dairy cattle (Ivie 1978; Van Niekerk 1994). In this outbreak, cows showed early signs of photosensitisation (teats and udder) as early as Day 3 of grazing the mixed forage crop. Such an early manifestation of clinical signs following introduction to a crop has been noted before (Collett 2014). As the turnips were the preferred feed of this crop, it would be reasonable to conclude that the turnips were responsible for hepatotoxicity. Furthermore, the animals had been introduced to the crop without any gradual transitioning to allow for rumen microbial adaptation. Hungry, pregnant and lactating cows tend to engorge over a short time, and this is most likely the reason for the acute toxicity.

In New Zealand, BALD in cows can be difficult to distinguish from FE (sporidesmin poisoning). The two diseases often occur at the same time of the year (late summer/autumn) and clinical signs of photosensitisation are indistinguishable (Collett & Matthews 2014). In both diseases, serum GGT activities can be severely elevated, reaching 10 or even 100 times the upper limit of the normal laboratory reference range (Collett 2014; Collett & Matthews 2014). Marked elevations in GGT activities within a few days of introduction to a crop (Day 5 in this outbreak) are not unusual (Bryan, Hea & Wilkinson 2015; Collett 2014). Similarly, in lambs dosed with sporidesmin, GGT activities begin to escalate within 2 days with a plateau at 10–14 days (Towers & Stratton 1978). These elevations potentially reflect necrosis, obstruction, proliferation, inflammation and/or neoplasia of the hepatobiliary-pancreatic duct system (Towers & Stratton 1978), even though morphological biliary changes, as in many BALD cases, may appear minimal (Collett & Matthews 2014). In the BALD outbreak reported here, the livers of Cases 13, 3 and 14 showed the loose, oedematous, concentric (onion skin) fibrosis around medium-sized bile ducts that is also seen in cases of FE. Other portal tract changes in these cows, such as inflammatory infiltrates and subtle changes in bile ducts were more non-specific.

Collett (2014) described lesions in small interlobular bile ducts in moribund cows with BALD that appeared to be of diagnostic value and that were distinctly different from those seen in sporidesmin toxicity. Since then, however, experience with a large outbreak of BALD in cows grazing swedes in New Zealand, where cows that suffered from photosensitisation were killed for welfare reasons, has shown that liver lesions, particularly regarding bile ducts, can be extremely subtle and varied, especially if the animal was not otherwise severely ill. Perhaps the severity or conspicuousness of liver lesions, especially those involving bile ducts, is dependent on the lethality of the toxicity? This underscores our lack of knowledge concerning most aspects of this toxicity, because most cases of BALD are not lethal and will recover if adequate alternative feed, supportive treatment and shade are provided (Collett & Matthews 2014).

Although the *Pithomyces* fungus does occur in some areas, FE is poorly reported in the South African veterinary literature. Published reports include cases in sheep in the Humansdorp district (Marasas et al. 1972) and in the winter rainfall area of the Western Cape Province and in cattle on a sewage farm near Johannesburg (Kellerman et al. 2005). However, farmers in the area (Humansdorp to Witelsbos in the Tsitsikamma district) are familiar with the condition and do see occasional cases of photosensitivity. The livers of cattle that have chronic FE are distinctive in that the ventral (left) lobe is smaller than normal as a result of atrophy, whilst the quadrate lobe is thicker as a result of regeneration (in sheep this deformation is often referred to as a ‘boxing glove’ liver; Kellerman & Coetzer 1985). On the other hand, BALD livers are mildly swollen but otherwise normal in shape (Collett 2014).

In 1997, a serious outbreak of cyanobacterial poisoning in Holstein cattle occurred near Humansdorp (Kellerman et al. 2005). The source of the poisoning was a water trough probably eutrophied by fertiliser. In this outbreak, cattle started dying within 24 h of drinking the water. Liver lesions in cyanobacterial poisonings comprise massive hepatic necrosis in peracute cases and fatty change and foci of necrosis with pigmentation of Kupffer cells in subacute cases (Kellerman et al. 2005).

A few *Crotalaria* spp. are known to be hepatotoxic as a result of the pyrrolizidine alkaloids that they contain (Kellerman et al. 2005). Sunhemp (*C. intermedia*), which was a very minor component of the crop, has low concentrations of these alkaloids in its seeds (Williams & Molyneux 1987). All parts of this plant, whether fresh or dried, however, should be regarded as toxic (Burrows & Tyrl 2013). Characteristic histological features of pyrrolizidine alkaloid hepatotoxicity include megalocytosis, nodular regeneration and fibrosis (Kellerman et al. 2005). The liver lesions in the BALD outbreak described here had no resemblance to those as a result of cyanobacteria or pyrrolizidine alkaloids.

It has been hypothesised that metabolism of high progoitrin concentrations in the turnips leads to nitrile derivatives of progoitrin that may be hepatotoxic (Matthews et al. 2020). The forage crop had been planted only 45 days ago. The maturity date for Barkant turnips is 70–90 days after sowing, so the crop was grazed whilst very immature. At 45 days, the plants would be mostly leaf, petiole and stem with very small bulbs, facilitating extremely rapid and easy harvest of soft leaf material by grazing cattle in contrast to a relatively slower rate of consumption of bulbs. Whilst progoitrin concentration is relatively lower in turnip leaves than bulbs, immature leafy turnip tops may, under conditions of rapid growth and in the presence of ample plant-available nitrogen and sulphur, accumulate progoitrin at levels sufficiently high to induce clinical BALD. Grazing immature turnips, therefore, would remain a major risk factor for BALD particularly when individual cattle consume turnips as a high proportion of total diet (C. Westwood [PGG Wrightson Seeds] New Zealand, pers. commun., 2021).

Because it is not possible, at present, to reproduce the disease in cattle, and therefore study lesion characteristics and pathogenesis, the diagnosis of BALD is dependent on whether the following conditions are present:

- History of sudden transitioning onto Barkant and English Giant varieties of *Brassica* without allowance for gradual rumen microbial adaptation
- Engorgement by hungry or dominant, often pregnant, cows
- Clinical signs of photosensitisation and/or jaundice in cows on a *Brassica* (turnip, rape or swede) crop
- Serum chemistry shows elevated activities of liver enzymes (GGT in particular)
- Enlarged yellowish-tan liver on gross necropsy examination
- Typical histological liver lesions (which may be subtle or even absent in the early stages of the condition), with characteristic abnormalities in small bile ducts, concentric peri-bile ductular fibrosis, mild portal fibrosis, cholestasis, and variable parenchymal necrosis
- Cessation of new cases within a few days of the cows being taken off the crop and provided with alternative feed
- In FE-prone areas, an up-to-date history of current *Pithomyces* spore counts on pastures that cows graze. Other aspects needing consideration include factors conducive to the growth of *Pithomyces*, such as time of the year, soil temperature, rainfall and/or irrigation, geographic location and availability of suitable substrate.

In South Africa, *B. rapa* ssp. *rapa* (turnip) crops have been used as fodder on numerous dairy farms for many years, seemingly without liver associated complications, however, the Barkant cultivar is a fairly recently introduced variety and reported to carry the highest risk for BALD. Rapid ingestion of large amounts of *Brassica* is a key determinant of toxicity, so it is essential that introduction to these crops be incremental and grazing must be strictly managed, preferably making use of strip grazing. Correct fodder flow planning, strict adherence to maturity times of different cultivars and stringent grazing management to accurately estimate the amount of *Brassica* fed per head per day as a proportion of the total diet are necessary to avoid potential toxicity (Westwood & Nichol 2009). To reduce the risk of toxicity, the recommendation is to first provide pasture, hay or silage so that cows have the opportunity to partially fill their rumens before beginning on approximately 2 kg *Brassica* dry matter/cow per day and then increase gradually to 5 kg dry matter/cow per day over the next 2 weeks (Collett & Matthews 2014). Special attention needs to be paid to ensure that dominant cows do not over engorge and that fences are secure.

In conclusion, we have described cases of photosensitivity in dairy cattle associated with the consumption of *Brassica* cover crops. This condition has been described in New Zealand for several years and has been termed BALD. This condition has not yet been described in the South African veterinary literature. Farmers and veterinarians need to be aware of the risks involved in feeding *Brassica* crops to cattle. Potentially, outbreaks of BALD could occur wherever forage *Brassica* crops are cultivated.
