# Corrigendum: Hepatogenous photosensitisation in cows grazing turnips (*Brassica rapa*) in South Africa

**DOI:** 10.4102/jsava.v92i0.2205

**Published:** 2021-09-17

**Authors:** Anthony J. Davis, Mark G. Collett, Johan C.A. Steyl, Jan G. Myburgh

**Affiliations:** 1 Humansdorp Veterinary Clinic, Humansdorp, South Africa; 2 School of Veterinary Science, Massey University, Palmerston North, New Zealand; 3 Department of Paraclinical Sciences, Faculty of Veterinary Science, University of Pretoria, Onderstepoort, South Africa

In the original article published, Davis, A.J., Collet, M.G., Steyl, J.C.A. & Myburgh, J.G., 2021, ‘Hepatogenous photosensitisation in cows grazing turnips (*Brassica rapa*) in South Africa’, *Journal of the South African Veterinary Association* 92(0), a2106. https://doi.org/10.4102/jsava.v92i0.2106, an author name was incorrectly spelt as Mark G. Collet. The correct spelling is Mark G. Collett. The authors apologise for this error. The correction does not change the study’s findings of significance or overall interpretation of the study’s results or the scientific conclusions of the article in any way.

